# Glucosamine/platelet-rich plasma/bone marrow MSC–loaded GelMA hydrogel supports cartilage endplate repair in mice and is associated with reduced inflammation- and oxidative stress–related readouts

**DOI:** 10.3389/fphar.2026.1819584

**Published:** 2026-06-15

**Authors:** Yinghao Bao, Baoyang Hu, Yang Bai, Ying Zhang, Hanlu Meng, He Wang, Fang Fang

**Affiliations:** 1 Spinal Surgery, Tongliao People’s Hospital, Tongliao, Inner Mongolia, China; 2 Department of Hands, Feet and Ankles, Tongliao People’s Hospital, Tongliao, Inner Mongolia, China

**Keywords:** bone marrow mesenchymal stem cells, cartilage endplate, GelMA, glucosamine, platelet-rich plasma

## Abstract

**Objective:**

In this study, we developed a gelatin methacryloyl (GelMA) hydrogel composite for the simultaneous delivery of glucosamine (GlcN), platelet-rich plasma (PRP), and bone marrow–derived mesenchymal stem cells (BMMSCs). We systematically investigated its effects on cartilage endplate (CEP) cell behavior, inflammation, and oxidative stress, and evaluated its reparative efficacy in a murine caudal CEP injury model.

**Methods:**

GlcN/PRP/BMMSC@GelMA was fabricated and comprehensively characterized in terms of its microarchitecture, compressive and rheological properties, enzymatic degradability, swelling, and the sustained release profile of GlcN and platelet-derived growth factor (PDGF). Additionally, primary CEP cells were treated with either GelMA or GlcN/PRP/BMMSC@GelMA to evaluate cell viability, proliferation, apoptosis, and the expression of chondrogenic markers (COL2A1, ACAN, and SOX9) using RT-qPCR and Western blot. Under lipopolysaccharide (LPS) challenge, cytokines (IL-1β, IL-6, TNF-α, IL-10) were quantified using ELISA. Under H_2_O_2_ exposure, reactive oxygen species (ROS) levels and oxidative stress markers (MDA, SOD, GSH) were assessed. *In vivo*, male BALB/c-nu nude mice underwent caudal disc puncture at the Co5/6 level using a 30G needle, followed by intradiscal injection of either GelMA or GlcN/PRP/BMMSC@GelMA. Tissue analyses were performed at 8 weeks post-procedure.

**Results:**

The composite formed a stable three-dimensional network with higher elastic and storage moduli than GelMA alone and supported the sustained release of GlcN and PDGF. The composite also improved CEP cell viability and proliferation, reduced apoptosis, and increased the expression of collagen II, aggrecan, and SOX9. In addition, it reduced LPS-induced inflammatory cytokines and mitigated H_2_O_2_-induced oxidative stress–related changes *in vitro*. *In vivo*, treatment was associated with improved CEP structural continuity, higher cartilage-related marker expression, and favorable inflammation- and oxidative stress–related readouts.

**Conclusion:**

GlcN/PRP/BMMSC@GelMA showed favorable *in vitro* and *in vivo* effects and supports the therapeutic potential of this combined local delivery platform for CEP repair.

## Introduction

Low back pain (LBP) is defined as pain or discomfort localized in the lumbar region. While most individuals experience at least one episode of LBP during their lifetime, a subset of patients develops persistent symptoms that may progress to chronic LBP. Global analyses conducted across 195 countries have identified LBP as a leading contributor to disability and productivity loss, thereby underscoring its significant public health burden (Knezevic et al., 2021). Intervertebral disc degeneration (IVDD) is a key structural correlate of LBP and is characterized by pathological changes in the nucleus pulposus, annulus fibrosus, and cartilage endplate (CEP) (Feng et al., 2025). CEP is a thin layer of hyaline cartilage that separates the vertebral body from the nucleus pulposus (NP) and is critical for nutrient transport between the vertebral marrow and the avascular disc. Accumulating evidence suggests that structural disruption and functional impairment of the CEP are closely associated with the initiation and progression of IVDD (Wang et al., 2024).

Pathological alterations of CEP—including microfractures, calcification, and the ingrowth of vascular or fibrous tissue—may impair nutrient transport to NP, thereby disrupting disc homeostasis (Han et al., 2019). Oxidative stress and inflammation have emerged as pivotal factors in the pathogenesis of CEP degeneration (Yang et al., 2024). Excessive production of reactive oxygen species (ROS) compromises cellular proteins, lipids, and DNA, ultimately precipitating cellular dysfunction and death (Sun et al., 2021). Concurrently, inflammatory signaling amplifies local tissue injury while promoting extracellular matrix degradation and apoptosis via multiple downstream pathways (McGarry et al., 2018). Collectively, these processes accelerate CEP degeneration and establish a self-perpetuating cycle linking CEP degeneration, nutrient deficiency, and IVDD (Ma et al., 2024). Accordingly, restoring CEP function and enhancing the disc’s nutritional microenvironment have emerged as promising therapeutic strategies for preventing—and potentially slowing or reversing—the progression of IVDD (Wong et al., 2019).

In recent years, biologically based interventions have attracted increasing attention for treating cartilage and intervertebral disc degeneration, encompassing small-molecule agents, bioactive factors, stem/progenitor cell therapies, and tissue-engineered scaffolds (Leu Alexa et al., 2021). Glucosamine (GlcN) is a critical precursor in glycosaminoglycan biosynthesis and has been extensively studied for its role in cartilage protection. Evidence suggests that GlcN supports chondrocyte anabolic metabolism while mitigating the expression of pro-inflammatory mediators (Suo et al., 2020; Chang et al., 2023). Platelet-rich plasma (PRP), which contains various growth factors, has emerged as a promising biologic therapy with potential reparative and anti-inflammatory effects in the treatment of cartilage injury (Kennedy et al., 2018; Collins et al., 2021). Bone marrow–derived mesenchymal stem cells (BMMSCs) exhibit multilineage differentiation capabilities and robust immunomodulatory properties, establishing them as a vital cellular resource in contemporary cartilage regeneration research (Salonius et al., 2020; Ivanovska et al., 2025).

Recent advancements in tissue engineering and biomaterials have spurred significant interest in the co-delivery of drugs, growth factors, and stem cells using three-dimensional (3D) scaffold-based composites. Among these platforms, hydrogels have emerged as a cornerstone of cartilage tissue engineering due to their superior biocompatibility, adjustable mechanical properties, and 3D that effectively mimic the essential characteristics of the extracellular matrix (Dong et al., 2019; Naolou et al., 2025). Gelatin methacryloyl (GelMA) is a photocrosslinkable hydrogel synthesized through the methacrylation of gelatin, which combines gelatin’s intrinsic bioactivity with tunable physicochemical properties. This hydrogel provides a conducive three-dimensional microenvironment for BMMSCs adhesion, proliferation, and differentiation. Furthermore, GelMA serves as a local delivery matrix for the sustained release of small-molecule agents and PRP-derived bioactive factors, thereby extending local bioavailability and enhancing therapeutic efficacy (Miri et al., 2018).

Building on our optimized GelMA formulation and component concentrations, we engineered a composite hydrogel incorporating GlcN, PRP, and BMMSCs. We systematically evaluated its impact on CEP cells by examining cell proliferation, phenotypic maintenance, as well as anti-inflammatory and antioxidant responses. Furthermore, the *in vivo* reparative potential of the composite hydrogel was assessed using a mouse model of coccygeal puncture–induced CEP injury. Collectively, our findings provide experimental evidence to support biomaterial-based strategies for the treatment of CEP injury and intervertebral disc degeneration.

## Materials and methods

### Main experimental materials, reagents and antibodies

Cells and biomaterials: Mouse BMMSCs (CP-M131) and complete medium (CM-M131) were purchased from Wuhan Ponsure Life Science and Technology Co., Ltd (Wuhan, China). Mouse CEP cells (SNL-178) and the corresponding specialized medium (SNLM-178) were obtained from Shanghai Shangen Biotechnology Co., Ltd (Shanghai, China). GelMA powder was synthesized in-house. GlcN (HY-B1125) was purchased from MedChemExpress (Monmouth Junction, NJ, United States). PRP was prepared from whole blood collected from donor mice of the same species.

Experimental animals: Male BALB/c-nu mice (6–8 weeks old; 18–20 g) were purchased from Hebei Taikang Medical Testing Services Co., Ltd. The animal use license number was SYXK (Ji) 2021-006. All animal experiments were conducted in accordance with the ARRIVE guidelines. Mice were housed under specific pathogen-free conditions at 22 °C ± 2 °C with 50%–60% relative humidity and a 12 h light/12 h dark cycle, with free access to food and water.

Reagents used in this study included glucosamine (GlcN; MedChemExpress, Monmouth Junction, NJ, United States; HY-B1125), lipopolysaccharide (LPS; MedChemExpress, Monmouth Junction, NJ, United States; HY-D1056), and hydrogen peroxide (H_2_O_2_; Merck, Darmstadt, Germany; 88597). ELISA kits for IL-1β, IL-6, TNF-α, and IL-10 were purchased from Medical Discovery Leader (Beijing, China) (catalog nos. MD6758, MD123475, MD7125, and MD132596, respectively). Commercial assay kits for superoxide dismutase (SOD), malondialdehyde (MDA), and glutathione (GSH) were purchased from Nanjing Jiancheng Bioengineering Institute (Nanjing, China) (A001-3, A006, and A003-1-2-1).

### The preparation of PRP

An anticoagulant vacuum blood collection vessel containing 5 mL of arterial blood was placed in a centrifuge and spun at 200 g for 10 min. Centrifugation separated the whole blood into three layers: serum, a white film, and red blood cells. In a sterile environment, the fluid above the erythrocyte layer was aspirated into a sterile centrifuge tube. After a subsequent centrifugation, the upper layer was platelet-poor plasma and the lower layer was platelet concentrate. Three-fourths of the supernatant was discarded, leaving the PRP.

### Screening of the action conditions of GlcN and PRP on CEP cells

CEP cells were cultured to ∼80% confluence, trypsinized with 0.25% trypsin and resuspended in complete medium containing 10% fetal bovine serum (FBS). Cells were seeded into 96-well plates (1 × 10^4^ cells/well) and incubated overnight at 37 °C in 5% CO_2_. For GlcN dose screening, cells were treated with GlcN at 0, 10, 25, 50, 100, and 200 μM, and viability was assessed using the CCK-8 assay at 24, 48 and 72 h. For PRP fraction screening, cells were treated with PRP at 0%, 5%, 10%, 15%, 20%, and 25% (v/v) under identical conditions, with viability measured at the same time points. Each condition included three technical replicate wells, and the experiments were repeated independently three times. According to the manufacturer’s protocol, 10 μL of CCK-8 reagent was added to each well and incubated for 1 h at 37 °C. Absorbance was measured at 450 nm using wells containing medium plus CCK-8 without cells as the blank.

### Preparation of GlcN/PRP/BMMSC@GelMA

GelMA was synthesized using a methacrylic anhydride modification method with minor adjustments based on commonly used GelMA synthesis procedures. Briefly, 1 g of gelatin was dissolved in 10 mL PBS (pH 7.4) at 50 °C to obtain a 10% (w/v) gelatin solution. Methacrylic anhydride (MA; 0.5 mL per 10 mL gelatin solution, 5% v/v relative to the gelatin solution volume) was added dropwise under vigorous stirring, and the reaction was allowed to proceed for 1 h at 50 °C. After the reaction, 10 mL PBS was added to dilute the solution. The reaction mixture was then dialyzed against deionized water using a 12–14 kDa molecular weight cutoff dialysis membrane for 7 days, with the water changed every 6 h to remove unreacted MA and small-molecule by-products. The dialyzed solution was freeze-dried to obtain porous GelMA and stored at −20 °C until use.

For hydrogel preparation, a photoinitiator stock solution was prepared by dissolving 0.05 g of photoinitiator in 20 mL PBS at 40 °C–50 °C for 15 min with intermittent shaking, resulting in a stock concentration of 0.25% (w/v). The solution was stored in the dark at 4 °C before use. GelMA was dissolved in PBS to obtain precursor solutions with the desired mass fractions (10%–40%, w/v), and the photoinitiator stock solution was added to prepare the GelMA precursor solution.

Based on the preliminary screening results, GlcN and PRP were incorporated into the precursor solution at the selected concentrations, and BMMSCs were added at a density of 1 × 10^7 cells/mL. The mixture was thoroughly blended and transferred into PDMS molds or culture wells. The GelMA-based precursor solution was then photocrosslinked under ultraviolet light using the same curing protocol for all GelMA-based hydrogel groups to form GlcN/PRP/BMMSC@GelMA.

The degree of methacrylation/functionalization of the synthesized GelMA and detailed photocrosslinking parameters, including exact light intensity, exposure duration, and light-to-sample distance, were not independently optimized in the present study. Their potential influence on hydrogel performance is therefore addressed in the Discussion and Limitations sections.

### Swelling property test of GlcN/PRP/BMMSC@GelMA

The prefabricated GelMA samples were weighed to obtain the initial mass W0. The samples were then immersed in PBS at 37 °C and removed at predetermined time points. After excess surface PBS was gently removed with filter paper, each sample was weighed and recorded as Wt. The swelling ratio was calculated using the following plain-text formula:

$$\text{Swelling ratio }\left(\%\right)=\left[\left(\text{Wt}-W0\right)/W0\right]\;\times 100.$$

Where W0 represents the initial mass of the GelMA sample, and Wt represents the swollen mass at each time point.

### *In vitro* degradation experiment of GlcN/PRP/BMMSC@GelMA

For the *in vitro* degradation assay, GelMA samples were first freeze-dried and weighed to obtain the initial dry mass W0. The samples were then immersed in PBS containing 1 U/mL type II collagenase and incubated on a shaker at 37 °C. Samples were collected on days 1, 3, 7, 14, 21, and 28, washed three times to remove residual collagenase, freeze-dried, and weighed again. The remaining dry mass at each time point was recorded as Wt. The degradation rate was calculated using the following plain-text formula:

$$\text{Degradation rate }\left(\%\right)=\left[\left(W0-\text{Wt}\right)/W0\right]\times 100.$$

Where W0 represents the initial dry mass of the GelMA sample before degradation, and Wt represents the remaining dry mass after degradation at each time point.

### Release of platelet-derived growth factor (PDGF) from platelet-rich PRP

The *in vitro* release of platelet-derived growth factor (PDGF) from PRP@GelMA was quantified by enzyme-linked immunosorbent assay (ELISA). Prefabricated GelMA samples containing PRP were placed in 6-well plates, and 2 mL of DMEM was added to each well. Samples were incubated at 37 °C in a 5% CO2 atmosphere. Supernatants were collected from each well on days 1, 3, 7, 11, 14, and 21. After recording the collected volume, an equal volume of fresh DMEM was immediately added to maintain a constant volume, and all supernatants were stored at −80 °C until analysis. For measurement, frozen supernatants were thawed slowly at 4 °C and processed according to the ELISA kit instructions. Working standards were prepared, and blank, standard, and sample wells were established. Optical density (OD) was measured at 450 nm, and PDGF concentrations were determined from the OD–concentration standard curve. Cumulative release profiles were then plotted to assess PDGF release from GelMA.

### Release of GlcN and quantification by high-performance liquid chromatography

The *in vitro* release of GlcN from the hydrogels was quantified by high-performance liquid chromatography (HPLC). Hydrogels containing GlcN were placed in a 6-well plate and 2 mL of DMEM was added to each well. Plates were incubated at 37 °C with 5% CO_2_. Supernatants were collected on Days 1, 3, 7, 11, 14, and 21, and an equal volume of fresh medium was added; collected samples were stored at −80 °C.

A 5 μmol/L (5 μM) GlcN standard stock was prepared according to the molecular weight of GlcN and serially diluted in PBS (pH 8.5) to yield working standards of 2.5 μM, 1 μM, 500 nM, 100 nM, 10 nM, and 1 nM. Fmoc-NHS was dissolved in methanol to make a 2 mmol/L stock solution. Prior to analysis, sample supernatants were diluted 10-fold with PBS (pH 8.5). For derivatization, 0.2 mL of each diluted sample or standard was mixed with 0.4 mL of the Fmoc-NHS stock solution, reacted at 40 °C for 1 h, cooled, and then analyzed by HPLC.

### Rheology and compressive mechanics

Disc-shaped hydrogels (diameter 25 mm, height 1 mm) were prepared. After equilibration in PBS for 48 h, a frequency sweep was performed at 25 °C with a shear strain of 5% and an angular frequency range of 0.1–100 rad/s using a rotational rheometer to determine the storage modulus G′ and the loss modulus G''.

For compression testing, cylindrical hydrogels (diameter 4 mm, height 5 mm; n = 3) were prepared. After equilibration in PBS for 48 h, samples were compressed at 0.02 mm/s on a universal materials testing machine. Stress–strain curves were recorded, and the elastic modulus was determined from the linear region between 0% and 20% strain.

### Scanning electron microscope

After freeze-drying, the hydrogel was mounted on a copper stage with conductive adhesive, sputter-coated with gold for 60 s, and its surface and cross-sectional pore morphologies were imaged at 5 kV.

### Effects of GlcN/PRP/BMMSC@GelMA on CEP cells

Cells were allocated to three groups (n = 3): blank control, GelMA, and GlcN/PRP/BMMSC@GelMA. After overnight adhesion, the appropriate hydrogels were added and co-incubated for 48 h. The following assays were then performed: CCK-8 for cell viability; Annexin V-FITC/PI flow cytometry for early and late apoptosis rates; EdU immunofluorescence for proliferation; RT-qPCR for mRNA levels of Actin, Collagen II, Aggrecan, and SOX-9; and Western blot for protein levels of Collagen II, Aggrecan, SOX-9, and β-actin.

### Anti-inflammatory experiment

Samples were allocated to four groups (n = 4): blank control, LPS (5 μg/mL LPS for 12 h), LPS + GelMA (5 μg/mL LPS for 12 h followed by GelMA hydrogel for 48 h), and LPS + GlcN/PRP/BMMSC@GelMA (5 μg/mL LPS for 12 h followed by GlcN/PRP/BMMSC@GelMA for 48 h). After treatment, supernatants were collected, and IL-1β, IL-6, TNF-α, and IL-10 levels were measured by ELISA.

### Antioxidant experiment

Cells were assigned to four groups (n = 4): blank control, H_2_O_2_ (250 μM for 24 h), H_2_O_2_ + GelMA (GelMA added 48 h after the 24-h H_2_O_2_ exposure), and H_2_O_2_ + GlcN/PRP/BMMSC@GelMA (GlcN/PRP/BMMSC@GelMA added 48 h after the 24-h H_2_O_2_ exposure). Intracellular reactive oxygen species (ROS) were measured with a fluorescent probe. Superoxide dismutase (SOD) activity and malondialdehyde (MDA) content were also determined to assess oxidative stress.

### RT-qPCR and western blot

For RT-qPCR, total RNA was extracted with TRIzol, and purity was assessed by nucleic acid concentration (A_260_/A_280_ 1.8–2.1). cDNA was synthesized by reverse transcription, and relative expression was calculated by the 2^−^ΔΔCt method with β-actin as the internal reference.

For Western blot, protein extraction, BCA quantification, SDS-PAGE electrophoresis, wet membrane transfer, primary and secondary antibody incubation, and ECL development were performed following standard protocols. Bands were imaged using a chemiluminescence system, and gray-scale analysis was conducted.

### *In vivo* experiments

Male BALB/c-nu mice were randomly assigned to four groups (n = 8): blank control, injury, GelMA, and GlcN/PRP/BMMSC@GelMA. After acclimation, and under anesthesia with X-ray guidance, a 30 G needle was inserted approximately 1.5 mm into the Co5/6 intervertebral disc from the lateral side, rotated 180°, and held for 10 s to create the injury model. In the GelMA and GlcN/PRP/BMMSC@GelMA groups, the respective hydrogels were injected into the discs before needle withdrawal. The blank group received no treatment, and the injury group underwent puncture only, without hydrogel injection. Mice were maintained under standard conditions after surgery, euthanized 8 weeks later, and their caudal tissues were harvested.

Harvested caudal tissues were fixed in 4% paraformaldehyde for 24 h, decalcified for 1 month, dehydrated through graded ethanol, cleared in xylene, and paraffin-embedded. Serial paraffin sections (4 μm) were prepared, mounted on glass slides, dried, deparaffinized, and rehydrated. H&E staining was performed using standard procedures. Histological evaluation focused on endplate continuity, cellular organization, and matrix appearance, and representative images were captured under a light microscope (Hu et al., 2024).

Portions of the tissues were reserved for RT-qPCR and Western blot analyses to quantify Collagen II, Aggrecan, and SOX-9 expression. Additional tissue samples or serum were assayed for SOD, MDA, GSH, CAT, IL-1β, IL-6, TNF-α, and IL-10, and reactive oxygen species (ROS) staining was performed.

### Statistical analysis

Data are reported as mean ± SEM. All analyses were performed using GraphPad Prism 6. Before applying one-way ANOVA, the assumptions of independence of observations, normality, and homogeneity of variances were evaluated. Independence was ensured by the study design, in which different samples/animals were analyzed as independent groups. Normality was assessed using the Shapiro–Wilk test, and homogeneity of variances was evaluated using the Brown–Forsythe test. For datasets meeting these assumptions, one-way ANOVA followed by appropriate *post hoc* multiple-comparison testing was used. For pairwise comparisons, an unpaired t-test was applied. A *P* value <0.05 was considered statistically significant.

## Results

### Preparation and physicochemical properties of GlcN/PRP/BMMSC@GelMA

**FIGURE 1:**
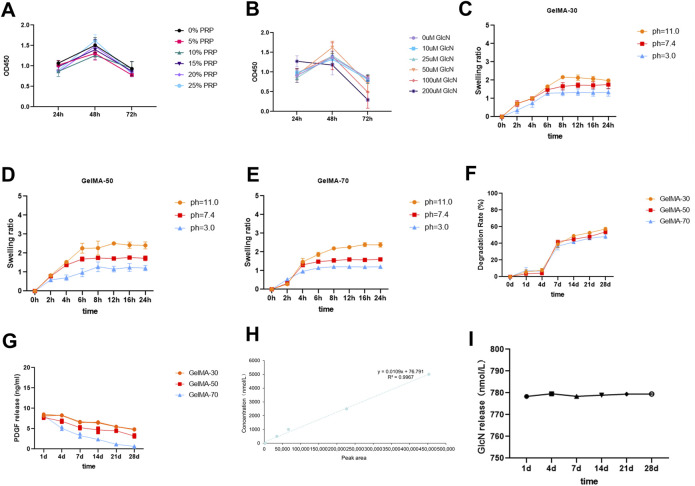
Screening of bioactive component doses and physicochemical characterization of GelMA hydrogels. **(A)** Cell metabolic activity (CCK-8; OD450) following treatment with increasing PRP proportions (0%–25%) for 24, 48, and 72 h. **(B)** Cell metabolic activity (CCK-8; OD450) following treatment with increasing GlcN concentrations (0–200 μM) for 24, 48, and 72 h. **(C–E)** Swelling kinetics of GelMA-30 **(C)**, GelMA-50 **(D)**, and GelMA-70 **(E)** under acidic (pH 3.0), neutral (pH 7.4), and alkaline (pH 11.0) conditions over 24 h. **(F)** *In vitro* degradation profiles of GelMA-30/50/70 over 28 days. **(G)** PDGF release profiles from GelMA-30/50/70 over 28 days. **(H)** Calibration curve used for GlcN quantification (y = 0.0109x + 76.791, *R* ^2^ = 0.9967). **(I)** GlcN release profile over 28 days determined using the calibration curve in **(H)**.

Based on prior screening, the final GlcN concentration in GlcN/PRP/BMMSC@GelMA was set to 50 μmol/L, and the PRP content to 25% (v/v) (Figures 1A,B). Swelling, *in vitro* enzymatic degradation, and release assays collectively indicated that the composite hydrogel maintained good structural stability and exhibited reproducible *in vitro* performance. In swelling assays, GelMA showed rapid PBS uptake at early time points, followed by a slower increase that approached a plateau; similar time-dependent profiles were observed under acidic and alkaline conditions (Figures 1C–E). Enzymatic degradation tests showed largely comparable degradation kinetics across GelMA concentrations over the observation period, without marked divergence (Figure 1F). PDGF release from GelMA-30 persisted throughout the study, supporting a sustained-release behavior (Figure 1G). Quantification by PDA scanning using a standard curve showed that GlcN levels remained ∼7.7 × 10^−7^ mol/L between days 21 and 28, supporting a relatively stable, sustained release profile (Figures 1H,I).

All GelMA-based hydrogels in the physicochemical characterization experiments were prepared using the same photocrosslinking protocol. In the preliminary formulation screening, GelMA-30, GelMA-50, and GelMA-70 referred to GelMA formulations with different nominal GelMA concentrations. Therefore, the observed differences in swelling, enzymatic degradation, and release behavior were interpreted mainly in relation to the GelMA formulation under a fixed photocrosslinking condition. GelMA concentration and crosslinking-related parameters may influence hydrogel network density, water uptake, enzymatic degradation, and molecular diffusion. Among the tested formulations, GelMA-30 showed a favorable balance between swelling behavior, degradation profile, and sustained PDGF release. Therefore, GelMA-30 was selected as the carrier matrix for subsequent GlcN/PRP/BMMSC@GelMA construction.

**FIGURE 2:**
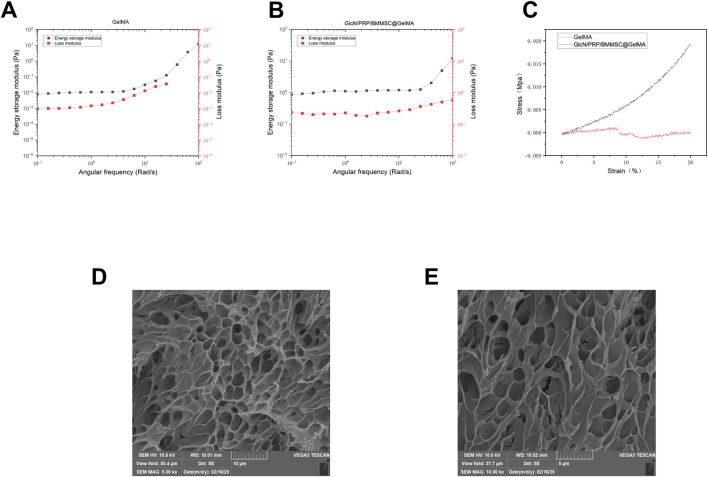
Mechanical properties and microarchitecture of the GlcN/PRP/BMMSC@GelMA hydrogel. **(A,B)** Oscillatory rheology frequency-sweep results for GelMA **(A)** and GlcN/PRP/BMMSC@GelMA **(B)**, showing the energy storage modulus (G′) and loss modulus (G″) as a function of angular frequency. **(C)** Compressive stress–strain curves comparing GelMA and GlcN/PRP/BMMSC@GelMA. **(D,E)** Representative SEM images of the porous microstructure of freeze-dried GelMA **(D)** and GlcN/PRP/BMMSC@GelMA **(E)**. Scale bars: 10 μm **(D)** and 5 μm **(E)**.

In rheological testing, GlcN/PRP/BMMSC@GelMA consistently showed G′ > G″ across the measured frequency range, indicating a viscoelastic material dominated by elastic behavior (Figures 2A,B). Compression tests revealed that the composite hydrogel produced higher stress than blank GelMA at the same strain. From the slope of the stress–strain curve in the 0%–10% strain range, the elastic modulus of GlcN/PRP/BMMSC@GelMA was 6.16 × 10^−4^ MPa, compared with 3.25 × 10^−5^ MPa for GelMA (Figure 2C). Scanning electron microscopy showed that blank GelMA formed a uniform, porous, sponge-like network, whereas the composite hydrogel preserved a porous framework with denser pore walls and improved pore connectivity, which may offer a more favorable microstructural environment for cell adhesion and nutrient and metabolite exchange (Figures 2D,E).

### GlcN/PRP/BMMSC@GelMA promotes the survival of CEP cells and the maintenance of cartilage phenotype

**FIGURE 3:**
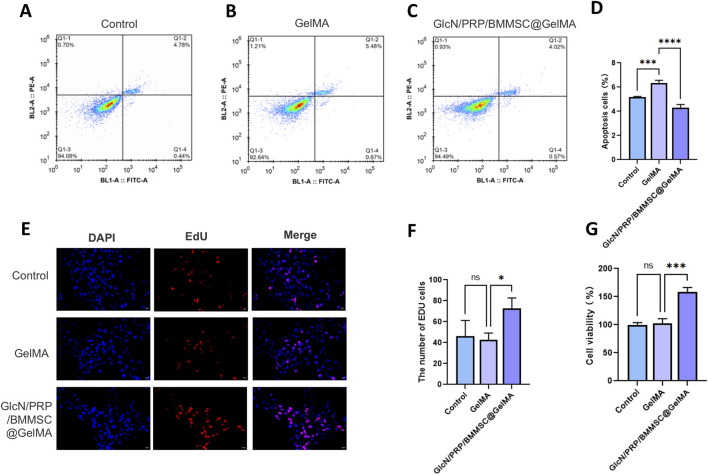
GlcN/PRP/BMMSC@GelMA enhances cell survival and proliferation while reducing apoptosis. **(A–C)** Representative flow cytometry plots of apoptosis in the control **(A)**, GelMA **(B)**,and GlcN/PRP/BMMSC@GelMA **(C)** groups. **(D)** Quantification of apoptotic cells (%) derived from the flow cytometry analysis. **(E)** Representative EdU staining images showing proliferating cells (EdU, red) and nuclei (DAPI, blue) in each group. **(F)** Quantification of EdU-positive cells. **(G)** Cell viability (%) in the indicated groups. * ns, not significant; *P < 0.05; ***P < 0.001; ****P < 0.0001.

CCK-8 assay (48 h) showed that CEP cell viability in the GelMA group did not differ significantly from that in the control group (ns), indicating no obvious cytotoxicity of GelMA. By contrast, viability in the GlcN/PRP/BMMSC@GelMA group increased to approximately 158%, a value that was significantly higher than in the GelMA group (***, *P* < 0.001). These results suggest that the composite formulation is more supportive of CEP cell viability and survival than GelMA alone under the present experimental conditions (Figure 3G).

Annexin V-FITC/PI flow cytometry showed that the control group had 94.08% viable cells (Annexin V^−^/PI^−^, Q1-3), 0.44% early apoptotic cells (Annexin V+/PI^−^, Q1-4), and 4.78% late apoptotic/necrotic cells (Annexin V+/PI+, Q1-2). In the GelMA group, viable cells fell to 92.64%, early and late apoptotic/necrotic cells were 0.67% and 5.48%, respectively, and the total apoptosis rate (Q1-4 + Q1-2) rose from 5.22% to 6.15%. By contrast, the GlcN/PRP/BMMSC@GelMA group showed 94.49% viable cells, 0.57% early apoptotic cells, 4.02% late apoptotic/necrotic cells, and a total apoptosis rate of 4.59%. Quantitative statistics matched these trends: the GelMA group had a higher apoptosis rate than the control group (***, *P* < 0.001), while the GlcN/PRP/BMMSC@GelMA group had a significantly lower rate than the GelMA group (****, *P* < 0.0001), indicating that the composite material reduced apoptosis under GelMA treatment conditions (Figures 3A–D). EdU staining showed that the proportion of EdU-positive cells in the GelMA group was similar to that in the control group, with no statistically significant difference (ns). In contrast, the GlcN/PRP/BMMSC@GelMA treatment produced a marked increase in EdU-positive cells; quantitative analysis confirmed that this increase was significantly higher than in the GelMA group (*, *P* < 0.05), indicating that the composite material promotes CEP cell proliferative activity (Figures 3E,F).

**FIGURE 4:**
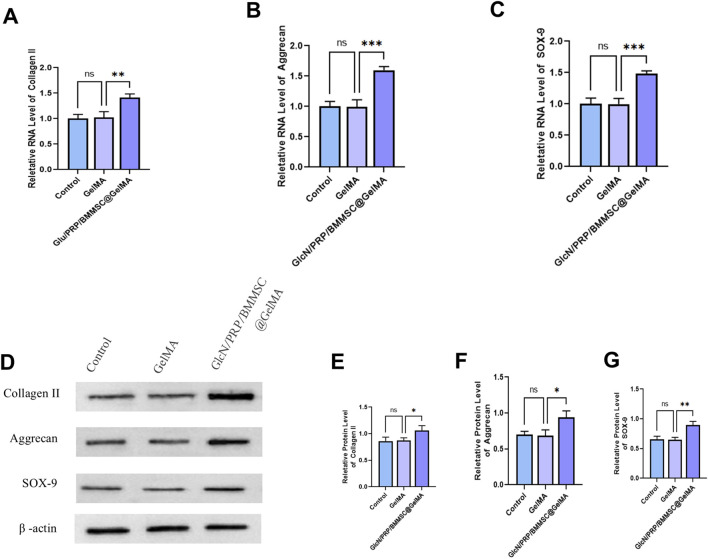
GlcN/PRP/BMMSC@GelMA promotes chondrogenic/anabolic marker expression. **(A–C)** RT-qPCR analysis of COL2A1 (Collagen II) **(A)**, ACAN (Aggrecan) **(B)**, and SOX9 **(C)** mRNA levels in the indicated groups (Control, GelMA, and GlcN/PRP/BMMSC@GelMA). **(D)** Representative Western blot bands for Collagen II, Aggrecan, and SOX-9, with β-actin as the loading control. **(E–G)** Densitometric quantification of protein expression for Collagen II **(E)**, Aggrecan **(F)**, and SOX-9 **(G)**, normalized to β-actin. * ns, not significant; *P < 0.05; **P < 0.01; ***P < 0.001.

RT-qPCR analysis showed that, compared with the control group, the GelMA group exhibited no significant changes (ns) in mRNA levels of COL2A1 (Collagen II), ACAN (Aggrecan), or SOX9. In contrast, treatment with GlcN/PRP/BMMSC@GelMA significantly upregulated these cartilage phenotype–related genes: COL2A1 rose by ∼1.4-fold (**, *P* < 0.01), ACAN by ∼1.6-fold (***, *P* < 0.001), and SOX9 by ∼1.5-fold (***, *P* < 0.001) (Figures 4A–C). These findings indicate that the complex enhances expression of genes associated with a chondrocyte-like phenotype in CEP cells. Western blot results mirrored the RT-qPCR trends. Protein levels of Collagen II, Aggrecan, and SOX-9 in the GlcN/PRP/BMMSC@GelMA group were significantly higher than those in the control and GelMA groups. No significant differences were observed between the GelMA group and the control group (ns). In contrast, all three proteins were elevated in the GlcN/PRP/BMMSC@GelMA group; Collagen II and Aggrecan were significantly upregulated relative to GelMA (*, *P* < 0.05), and SOX-9 showed a further significant increase (**, *P* < 0.01) (Figures 4D–G). The β-actin band was stable, indicating consistent loading. These results suggest that the complex enhances expression of proteins associated with the chondrocyte-like phenotype of CEP cells.

### Anti-inflammatory effect of GlcN/PRP/BMMSC@GelMA

**FIGURE 5:**
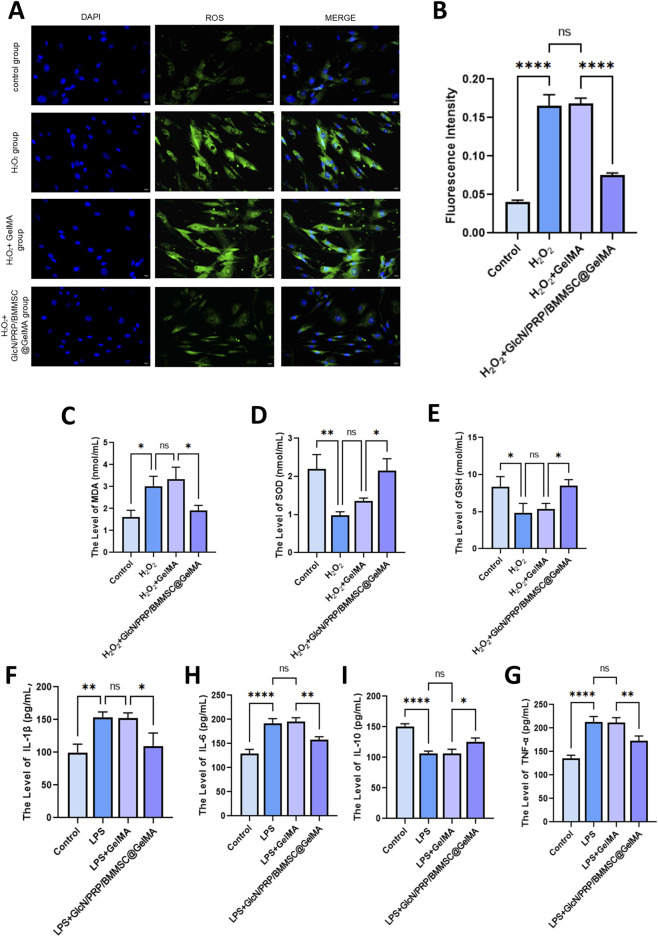
GlcN/PRP/BMMSC@GelMA attenuates oxidative stress and modulates inflammatory cytokine production *in vitro*. **(A)** Representative fluorescence images of intracellular ROS (green) with nuclear counterstaining (DAPI, blue) in the indicated groups after H_2_O_2_ challenge (Control, H_2_O_2_, H_2_O_2_+GelMA, and H_2_O_2_+GlcN/PRP/BMMSC@GelMA). **(B)** Quantification of ROS fluorescence intensity from **(A)**. **(C–E)** Biochemical assessment of oxidative stress–related indices, including MDA **(C)**, SOD **(D)**, and GSH **(E)**, in the indicated groups after H_2_O_2_ stimulation. **(F–I)** ELISA quantification of inflammatory cytokines following LPS stimulation, including IL-1β **(F)**, IL-6 **(H)**, IL-10 **(I)**, and TNF-α **(G)**, in the indicated groups (Control, LPS, LPS + GelMA, and LPS + GlcN/PRP/BMMSC@GelMA). ns, not significant; *P < 0.05; **P < 0.01; ****P < 0.0001.

An inflammatory response was induced by LPS, and cytokine concentrations in the supernatant were measured by ELISA. Compared with the control group, LPS stimulation produced a significant increase in IL-1β (**, *P* < 0.01), IL-6 (****, *P* < 0.0001), and TNF-α (****, *P* < 0.0001), and a significant decrease in IL-10 (****, *P* < 0.0001), indicating successful activation of inflammation. No significant differences were observed in IL-1β, IL-6, TNF-α, or IL-10 between the LPS group and the LPS + GelMA group (ns). Compared with the LPS + GelMA group, the LPS + GlcN/PRP/BMMSC@GelMA group showed reduced levels of IL-1β (*, *P* < 0.05), IL-6 (**, *P* < 0.01), and TNF-α (**, *P* < 0.01), and an increased level of IL-10 (*, *P* < 0.05). These results indicate that the composite material downregulates proinflammatory cytokines and upregulates the anti-inflammatory cytokine IL-10 under LPS-induced conditions, consistent with an anti-inflammatory regulatory effect (Figures 5F–I).

### Antioxidant effect of GlcN/PRP/BMMSC@GelMA

In the H_2_O_2_-induced oxidative stress model, MDA, SOD, and GSH were measured to assess lipid peroxidation and antioxidant capacity (Figure 5). After H_2_O_2_ treatment, MDA increased significantly (*, *P* < 0.05), SOD decreased significantly (**, *P* < 0.01), and GSH decreased significantly (*, *P* < 0.05), indicating enhanced oxidative damage and compromised antioxidant defense. The H_2_O_2_ + GelMA group showed no significant differences in MDA, SOD, or GSH compared with the H_2_O_2_ group (ns). By contrast, H_2_O_2_ + GlcN/PRP/BMMSC@GelMA group exhibited a significant reduction in MDA (*, *P* < 0.05) and significant increases in both SOD and GSH (*, *P* < 0.05) relative to the H_2_O_2_ + GelMA group, indicating that this formulation attenuates H_2_O_2_-induced lipid peroxidation and restores cellular antioxidant capacity (Figures 5C–E).

ROS fluorescence imaging and quantitative analysis (Figures 5A,B) showed that intracellular ROS levels increased markedly after H_2_O_2_ treatment compared with the control group (****, *P* < 0.0001). ROS intensity did not differ significantly between the H_2_O_2_ + GelMA group and the H_2_O_2_ group (ns). By contrast, ROS intensity in the H_2_O_2_ + GlcN/PRP/BMMSC@GelMA group was significantly reduced relative to the H_2_O_2_ + GelMA group (****, *P* < 0.0001), indicating that this composite effectively mitigates H_2_O_2_-induced intracellular ROS accumulation (Figures 5A,B).

### Repair effect of GlcN/PRP/BMMSC@GelMA in a mouse model of CEP injury

**FIGURE 6:**
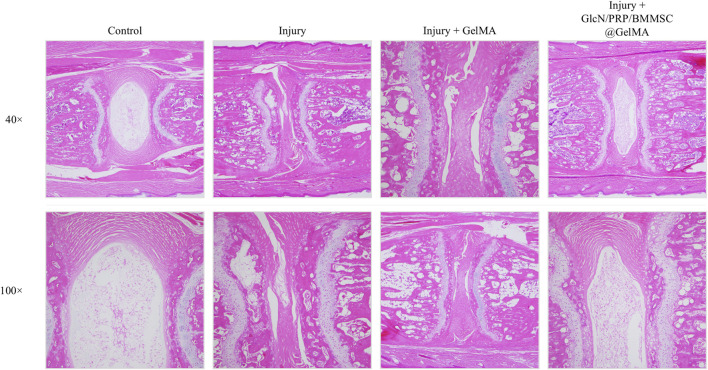
Histological evaluation of caudal tissues after injury and hydrogel treatment *in vivo*. Representative hematoxylin and eosin (H&E)–stained sections of the caudal disc/endplate region from the control, injury, injury + GelMA, and injury + GlcN/PRP/BMMSC@GelMA groups. Images are shown at 40 × (top row) and 100 × (bottom row) magnification.

In the mouse caudal vertebral CEP injury model, H&E staining showed that the control group retained an intact endplate structure with relatively regular cell arrangement (Figure 6). The injury group exhibited disrupted endplate architecture, disorganized cellular arrangement, and reduced matrix. The GelMA group showed partial improvement relative to the injury group, but endplate continuity remained limited. In contrast, the GlcN/PRP/BMMSC@GelMA group showed better preservation of endplate continuity, more regular tissue architecture, and stronger matrix staining. These findings support morphological improvement in the repaired region, but they do not by themselves define the underlying immune-cell dynamics.

**FIGURE 7:**
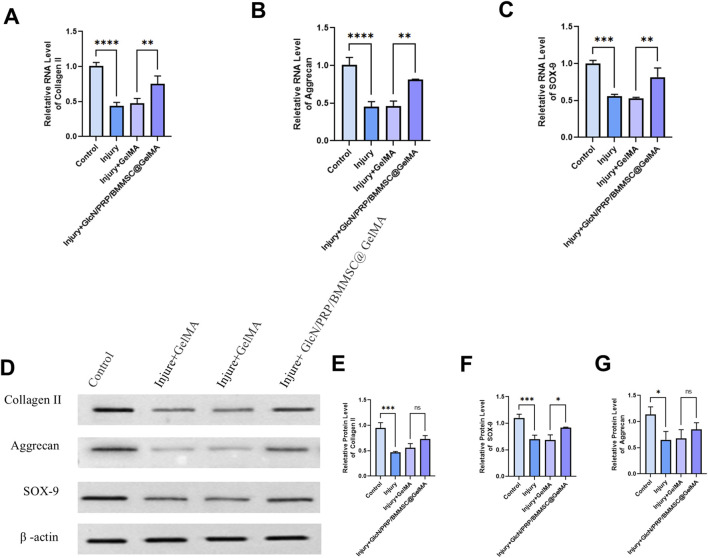
GlcN/PRP/BMMSC@GelMA restores cartilage-associated marker expression *in vivo*. **(A–C)** RT-qPCR analysis of COL2A1 (Collagen II) **(A)**, ACAN (Aggrecan) **(B)**, and SOX9 **(C)** in caudal tissues from the Control, Injury, Injury + GelMA, and Injury + GlcN/PRP/BMMSC@GelMA groups. **(D)** Representative Western blot bands for Collagen II, Aggrecan, and SOX-9, with β-actin as the loading control. **(E–G)** Densitometric quantification of protein expression for Collagen II **(E)**, SOX-9 **(F)**, and Aggrecan **(G)**, normalized to β-actin. ns, not significant; *P < 0.05; **P < 0.01; ***P < 0.001; ****P < 0.0001.

Molecular-level analyses (Figure 7) showed that, relative to the control group, mRNA levels of COL2A1 (Collagen II), ACAN (Aggrecan), and SOX9 were downregulated in the injury group. Treatment with GlcN/PRP/BMMSC@GelMA upregulated these genes, and their mRNA levels were higher than those in the injury + GelMA group (Figures 7A–C). Western blot results paralleled the RT-qPCR trends (Figure 7D): following injury, protein levels of Collagen II, Aggrecan, and SOX-9 decreased, and these proteins recovered after GlcN/PRP/BMMSC@GelMA treatment (Figures 7D–G). A stable β-actin band confirmed consistent sample loading. Together, these findings indicate that the composite hydrogel supports the restoration of cartilage-associated molecular markers in the *in vivo* injury environment.

Additional biochemical analyses further supported favorable inflammation- and oxidative stress-related readouts after treatment (Supplementary Figure S1).

## Discussion

The integration of biomaterials with localized controlled-release therapy has recently emerged as a promising strategy for treating IVDD (Liu et al., 2023). Conventional pharmaceutical and surgical approaches offer limited benefits, whereas the targeted delivery of therapeutically validated agents via biomaterials has the potential to mitigate degeneration and injury of the CEP, ultimately enhancing IVDD treatment outcomes. In particular, a composite GelMA system loaded with GlcN, PRP, and BMMSCs may serve as a promising platform for CEP repair. As a photocrosslinkable biomaterial, GelMA exhibits high biocompatibility and biodegradability while providing a three-dimensional, biomimetic microenvironment conducive to cell proliferation and differentiation (Zhang et al., 2025).

The CEP is a crucial anatomical structure that links the vertebral body to the intervertebral disc. As the primary route for nutrient exchange, it maintains the metabolic homeostasis of the disc (Tang et al., 2024). Recent reviews have concluded that CEP degeneration is both an early event in IVDD and a driving factor in its pathological progression. Degenerated CEP releases inflammatory mediators (for example, IL-1β and TNF-α) and reactive oxygen species (ROS) (Tang et al., 2024). These agents activate signaling cascades such as NF-κB and MAPK, which increase the expression of extracellular matrix–degrading enzymes (for example, MMPs and ADAMTS), thereby accelerating nucleus pulposus degeneration. Collagen II, aggrecan, and SOX-9 are widely used core markers in cartilage research; they respectively represent the matrix structural framework, the compressive functional matrix, and the cartilage-lineage transcriptional program. Inflammation and oxidative stress are major triggers of CEP degeneration (Kamali et al., 2021; Zhang et al., 2021). IL-1β, IL-6, TNF-α, and IL-10 are commonly applied *in vitro* to mimic the inflammatory microenvironment of OA, and they serve as primary indicators for establishing inflammatory models or assessing anti-inflammatory effects. Measures such as ROS, MDA, and SOD reflect oxidative stress and antioxidant capacity, and they are routinely used as objective readouts of oxidative damage and the efficacy of interventions in both *in vitro* and *in vivo* models (Ragni et al., 2023).

GlcN, a natural amino monosaccharide, has demonstrated anti-inflammatory and anabolic effects in chondrocytes. Tian Xie et al. report that GlcN ameliorates mitochondrial dysfunction, extracellular matrix degradation, and inflammatory signaling by activating the Nrf2/HO-1 pathway and inhibiting the MAPK pathway, thereby delaying IVDD progression (Xie et al., 2024). According to Altalbawy et al., PRP contributes to tissue repair by releasing bioactive factors such as platelet-derived growth factor (PDGF) and vascular endothelial growth factor (VEGF); it also modulates inflammation by reducing M1 macrophage polarization and promotes matrix synthesis and angiogenesis via the PI3K/AKT/mTOR pathway. According to Altalbawy et al. further found that in IVDD therapy, MSCs both differentiate into chondrocyte-like cells and influence nucleus pulposus cells, annulus fibrosus, and CEP biology through secreted exosomes (for example, miRNAs), thereby inhibiting apoptosis and enhancing extracellular matrix synthesis (Uchiyama et al., 2021; Altalbawy et al., 2025).

Although the combined GlcN/PRP/BMMSC-loaded GelMA construct showed more favorable biological and reparative readouts than GelMA alone, the present study was designed as a proof-of-concept evaluation of the integrated platform rather than a factorial dissection study. Therefore, the relative contribution of GlcN, PRP, and BMMSCs could not be determined from the current dataset, and a synergistic interaction among the components should not be inferred. In particular, a dominant contribution from PRP-derived growth factors cannot be excluded. Future studies incorporating single-component and dual-component controls will be required to clarify component-specific effects and potential interactions.

GelMA is an injectable, photo-crosslinkable, gelatin-derived hydrogel that presents ECM-mimetic adhesion motifs and degrades in response to MMP activity. Its mechanical properties and microstructure (for example, porosity and degradation kinetics) are tunable via the degree of methacrylation, polymer concentration, and crosslinking conditions, making it a practical platform for co-delivering cells and bioactive cues to enhance local retention and tissue compatibility (Yue et al., 2015; Bupphathong et al., 2022). Systemic delivery of GlcN may fail to reach therapeutic concentrations at the target site, whereas incorporation of GlcN into GelMA permits localized, sustained exposure as the matrix slowly degrades (Wang et al., 2022; Chang et al., 2023). When combined with PRP, GelMA also functions as a growth-factor depot that attenuates burst release of key factors (for example, PDGF-BB and VEGF-A) and prolongs paracrine signaling to support repair. Concurrently, GelMA provides a three-dimensional microenvironment for BMMSCs that supports adhesion, spreading, and proliferation while allowing material properties to modulate cell-perceived mechanical cues (Li et al., 2021; Ramos-Rodriguez and Leach, 2023). In cartilage-related applications, GelMA has been employed to retain viable cells at cartilage surfaces or defect sites, underscoring its potential for endplate-side cell delivery and localized microenvironmental remodeling (Hölzl et al., 2022).

GelMA hydrogel performance is closely associated with photocrosslinking-related parameters and formulation variables, including the degree of methacrylation/functionalization, GelMA concentration, photoinitiator concentration, light wavelength/intensity, and exposure duration (Arguchinskaya et al., 2023; Shehzad et al., 2023). The degree of methacrylation/functionalization determines the number of photo-reactive groups available for network formation, whereas GelMA concentration and photocrosslinking conditions collectively regulate crosslinking density, pore architecture, swelling behavior, degradation rate, molecular diffusion, and mechanical stiffness. Therefore, the physicochemical behavior observed in the present study should be interpreted in the context of the standardized photocrosslinking protocol and the selected GelMA formulation.

In this study, the GlcN/PRP/BMMSC@GelMA hydrogel showed higher compressive stiffness than blank GelMA while preserving a porous microstructure, suggesting that incorporation of GlcN, PRP, and BMMSCs did not disrupt the basic GelMA network but instead generated a composite matrix with improved mechanical support and sustained-release capacity. Nevertheless, because the degree of methacrylation/functionalization, photoinitiator concentration, light intensity, and exposure duration were not independently varied, the present study cannot determine the individual contribution of each GelMA-related parameter to cartilage endplate repair.

Based on the above research, we prepared GelMA loaded with GlcN, PRP, and BMMSCs and performed a systematic evaluation of its physicochemical properties, *in vitro* cellular effects, and *in vivo* repair of CEP injury. GlcN/PRP/BMMSC@GelMA showed improved compressive mechanical properties, with an elastic modulus higher than that of blank GelMA, while preserving and optimizing a three-dimensional porous microstructure. These features confer enhanced structural stability, mechanical support, and a microenvironment favorable for material transport and cell compatibility. The composite also enabled delayed diffusion and sustained release of different payloads (growth factors and small molecules), supporting prolonged maintenance of local effective concentrations. *In vitro*, GlcN/PRP/BMMSC@GelMA markedly increased CEP cell viability, reduced apoptosis, raised the proportion of EdU-positive cells, and upregulated Collagen II, Aggrecan, and SOX-9 at both the gene and protein levels. These results indicate that the formulation improves CEP cell status across the axes of activity, proliferation, and phenotype maintenance, suggesting support for CEP cell phenotype maintenance and matrix-related expression. In the LPS-induced inflammation model, GlcN/PRP/BMMSC@GelMA reduced the proinflammatory cytokines IL-1β, IL-6, and TNF-α and increased the anti-inflammatory cytokine IL-10. In the H_2_O_2_-induced oxidative stress model, GlcN/PRP/BMMSC@GelMA decreased ROS and MDA while raising SOD and related markers. *In vivo*, GlcN/PRP/BMMSC@GelMA was associated with improved histological repair features and restoration of cartilage-associated molecular markers: the CEP architecture became more intact, matrix staining intensified, expression of cartilage-associated genes and proteins was restored, and measures of oxidative stress and inflammation showed measurable improvement.

### Limitations

This study has several limitations. First, it primarily relies on *in vitro* assays and a murine caudal injury model; therefore, the findings cannot be directly extrapolated to human degenerative disc disease. Second, although H&E staining demonstrated morphological improvement after treatment, cell-specific immunohistological analyses were not performed. As a result, the local reparative immune landscape, including neutrophil-, macrophage-, and mast cell–related responses, could not be comprehensively defined. Third, the present study did not include single-component or dual-component control groups; therefore, the relative contributions of GlcN, PRP, and BMMSCs, as well as possible synergistic interactions among them, remain unclear. Fourth, although the hydrogel was characterized in terms of swelling, degradation, release behavior, rheology, compression properties, and microstructure, GelMA-related parameters were not systematically optimized in a factorial manner. These parameters include the degree of methacrylation/functionalization, GelMA concentration, photoinitiator concentration, light wavelength, light intensity, exposure duration, and light-to-sample distance. Therefore, the independent and interactive effects of these parameters on hydrogel performance, cell behavior, tissue remodeling, and cartilage endplate repair remain to be clarified in future studies. In addition, quantitative histopathological or morphometric scoring was not performed in the present study, which limits the precision of lesion grading based on morphology alone. Future studies should address these issues in larger animal models and under more clinically relevant disc microenvironmental conditions.

## Conclusion

In summary, GlcN/PRP/BMMSC@GelMA showed favorable effects on CEP-related cell survival, cartilage-associated marker expression, and inflammation-/oxidative stress-related readouts, and was associated with improved repair features in a murine caudal CEP injury model. These findings support the potential of this combined GelMA-based delivery platform for CEP repair, while further studies are needed to define immune-cell responses, component-specific contributions, and translational applicability.

## Data Availability

The original contributions presented in the study are included in the article/Supplementary Material, further inquiries can be directed to the corresponding authors.
