# Supplementary material for: Glucosamine/platelet-rich plasma/bone marrow MSC–loaded GelMA hydrogel supports cartilage endplate repair in mice and is associated with reduced inflammation- and oxidative stress–related readouts
Source: Front Pharmacol. 2026 Jun 15;17:1819584. doi: 10.3389/fphar.2026.1819584 (PMC13312886; doi:10.3389/fphar.2026.1819584)
Supplement: Supplementary file 1 [file Supplementaryfile1.pdf]

Supplementary Material

Supplementary Figure S1

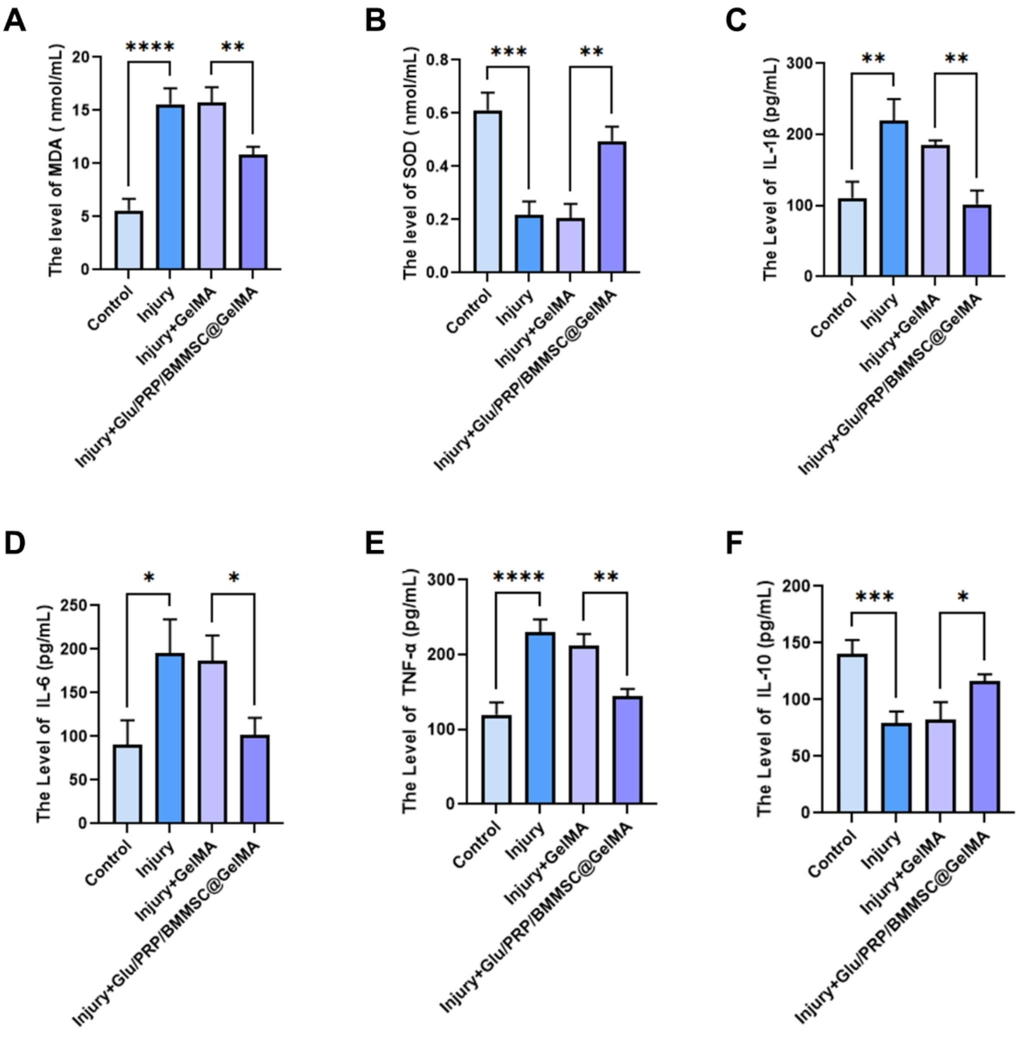

Supplementary Fig. S1. In vivo inflammation- and oxidative stress-related readouts after treatment. (A) MDA levels and (B) SOD activity in caudal endplate tissues from the control, injury, injury + GelMA, and injury + GlcN/PRP/BMMSC@GelMA groups.
(C–F) ELISA quantification of IL-1β, IL-6, TNF-α, and IL-10 in the same groups. ns, not significant; *$P < 0.05$; **$P < 0.01$; ***$P < 0.001$; ****$P < 0.0001$.
